# Co-regulation of mitochondrial respiration by proline dehydrogenase/oxidase and succinate

**DOI:** 10.1007/s00726-015-2134-7

**Published:** 2015-12-10

**Authors:** Chad N. Hancock, Wei Liu, W. Gregory Alvord, James M. Phang

**Affiliations:** Metabolism and Cancer Susceptibility Section, Basic Research Laboratory, Center for Cancer Research, National Cancer Institute, NCI-Frederick, 1050 Boyles Street, Bldg. 538, Rm. 144, Frederick, MD 21702-1201 USA; Data Management Services, National Cancer Institute at Frederick, Frederick, MD 21702 USA

**Keywords:** Respiration, Reactive oxygen species, Redox, Energetics, Signaling

## Abstract

Proline dehydrogenase/oxidase (PRODH/POX) is a mitochondrial protein critical to multiple stress pathways. Because of the roles of PRODH/POX in signaling, and its shared localization to the mitochondrial inner membrane with the electron transport chain (ETC), we investigated whether there was a direct relationship between PRODH/POX and regulation of the ETC. We found that PRODH/POX binds directly to CoQ1 and that CoQ1-dependent PRODH/POX activity required functional Complex III and Complex IV. PRODH/POX supported respiration in living cells during nutrient stress; however, expression of PRODH/POX resulted in an overall decrease in respiratory fitness. Effects on respiratory fitness were inhibited by DHP and NAC, indicating that these effects were mediated by PRODH/POX-dependent reactive oxygen species (ROS) generation. PRODH/POX expression resulted in a dose-dependent down-regulation of Complexes I–IV of the ETC, and this effect was also mitigated by the addition of DHP and NAC. We found that succinate was an uncompetitive inhibitor of PRODH/POX activity, inhibited ROS generation by PRODH/POX, and alleviated PRODH/POX effects on respiratory fitness. The findings demonstrate novel cross-talk between proline and succinate respiration in vivo and provide mechanistic insights into observations from previous animal studies. Our results suggest a potential regulatory loop between PRODH/POX and succinate in regulation of mitochondrial respiration.

## Introduction

Proline dehydrogenase (PRODH), a.k.a. proline oxidase (POX), is a mitochondrial inner membrane protein. Oxidation of proline to pyrroline-5-carboxylate (P5C) mediates the proline cycle that shuttles NADP(H)/NADP(+) redox equivalents between mitochondria and cytosol and forms a metabolic interlock with the pentose phosphate pathway (Hagedorn and Phang 1983; Phang 1985). Conversion of P5C to glutamine or ornithine also connects the proline cycle to the TCA and urea cycles, respectively.

PRODH/POX is a mediator of genotoxic, inflammatory, and metabolic stress signaling. Originally identified in a screen of p53-induced genes (Polyak et al. 1997; Campbell et al. 1997), induction of PRODH/POX by p53 was subsequently shown to result in mitochondrial-mediated apoptosis through generation of reactive oxygen species (ROS) in the form of mitochondrial superoxide (Donald et al. 2001; Hu et al. 2007; Liu et al. 2005). During hypoxia, tumor cells respond with either PRODH/POX-mediated ATP generation under conditions of low glucose, or pro-survival, ROS-mediated autophagy induction when glucose levels were normal (Liu et al. 2012). Activation of PRODH/POX by PPAR-γ results in PRODH/POX-dependent superoxide production that induces beclin-1 gene expression and activates protective autophagy versus the toxic effects of oxidized low-density lipoproteins (Zabirnyk et al. 2010). In adipocytes, PRODH/POX activation of PPAR-α through ROS-dependent and FOXO1-mediated induction prevents cell death and inflammation (Lettieri Barbato et al. 2014). In RKO colorectal cancer cells, induction of nutrient stress by either glucose withdrawal or treatment of cells with rapamycin results in increased AMP-activated protein kinase (AMPK)-dependent PRODH/POX catalytic activity, PRODH/POX-dependent cellular ATP generation, and activation of the pentose phosphate pathway (Pandhare et al. 2009). In *C. elegans*, AMPK-mediated upregulation of proline catabolism results in generation of ROS that promote an adaptive endogenous stress defense and increased lifespan (Zarse et al. 2012). Treatment of embryonic stem cells with proline induces their differentiation into either a primitive, ectoderm-type cell (Washington et al. 2010) or a novel, reversible epiblast stem cell-like state (Casalino et al. 2011). Thus, depending on cellular and environmental context, PRODH/POX can mediate programmed cell death, promote cell survival, or induce differentiation.

The mitochondrial respiratory electron transport chain (ETC) consists of a series of four protein complexes, known as Complexes I–IV, that pass electrons from the electron donors NADH and FADH through a series of redox reactions, creating an electrochemical proton gradient that is coupled with oxidative phosphorylation and the synthesis of ATP by ATP synthase (Chaban et al. 2014). Complex I, aka NADH dehydrogenase, passes electrons from NADH to reduce ubiquinone to ubiquinol. Complex II, aka succinate dehydrogenase (SDH), couples the oxidation of succinate to fumarate in the tricarboxylic acid (TCA) cycle with the reduction of ubiquinone to ubiquinol in the ETC (Hagerhall 1997). Electrons are transferred from this ubiquinol pool to Complex III, coenzyme Q-cytochrome C reductase, which passes the electrons to the hemeprotein cytochrome C. The final step is the transfer of electrons to Complex IV, cytochrome C oxidase, which passes the electrons to molecular oxygen to form water and drive the proton gradient that powers ATP synthase.

The SDH holoenzyme consists of four subunits, known as SDHA-D. SDHA contains the succinate binding site and flavin-adenine dinucleotide (FAD) redox center; SDHB contains three iron-sulfur centers required for electron transfer to ubiquinone, and SDHC and SDHD are two hydrophilic subunits responsible for anchoring the enzyme in the mitochondrial inner membrane. The ubiquinone-binding site of the holoenzyme is in a pocket formed by SDHB, SDHC, and SDHD at the surface of the matrix face of the mitochondrial inner membrane (Lancaster 2002; Horsefield et al. 2004; Sun et al. 2005). Mutations in SDHB, SDHC, and SDHD have been found in hereditary paraganglioma (Baysal et al. 2000; Niemann and Muller 2000; Astuti et al. 2001), gastrointestinal stromal tumors (GISTs) (Pasini et al. 2008), thyroid (Ricketts et al. 2010; Zantour et al. 2004), and renal tumors (Ricketts et al. 2008; Vanharanta et al. 2004). There is increasing evidence that mutations in SDH can lead to aberrant ROS generation through errors introduced in electron transport, and that this can result in tumorigenesis. ROS can be generated at the SDHA subunit during impaired electron transport (Yankovskaya et al. 2003). SDHC inactivation mutation in *C. elegans* and 3T3 mouse fibroblasts and electron leakage, oxidative stress, apoptosis and increased transformation and tumor growth (Adachi et al. 1998; Ishii et al. 2005).

Because of the pleiotropic role of PRODH/POX in cellular energetics and signaling, and its shared localization with the ETC on the inner membrane of the mitochondria, we sought to determine whether there was a direct relationship between PRODH/POX and regulation of the ETC. Using a PRODH/POX-expressing DLD colorectal cancer cell model and mouse mitochondria, we demonstrate that PRODH/POX passes electrons directly to Coenzyme Q1 (CoQ1), and that acute proline treatment in PRODH/POX-expressing cells resulted in Complex I- and Complex II-independent oxidative respiration during nutrient stress conditions. In contrast, exposure of cells to PRODH/POX and proline resulted in a significant time and dependent decrease in total oxidative respiration due to PRODH/POX-dependent ROS production. PRODH/POX had dose-dependent effect on the protein levels of individual subunits of Complexes I–IV of the ETC, which was reversed with the PRODH/POX inhibitor DHP and the antioxidant l-NAC. We show here that succinate inhibits PRODH/POX through uncompetitive inhibition, and treatment of cells with succinate inhibits production of PRODH/POX-dependent ROS, mitigates inhibition of respiration by PRODH/POX, and restores protein levels of ETC complexes in PRODH/POX-treated cells. These results suggest that PRODH/POX acts as a regulator of cellular respiration and that PRODH/POX activity is functionally linked to levels of succinate, potentially linking them as metabolic regulators.

## Materials and methods

### Chemicals and inhibitors

Rotenone, 2-thenoyltrifluoroacetone, antimycin A, myxothiazol, potassium cyanide, 3,4-dehydro-l-proline, carboxin, methyl-succinate, l-proline, coenzyme Q1, doxycycline, *N*-acetyl cysteine, and *o*-aminobenzaldehyde were purchased from Sigma Aldrich, St. Louis, MO. Atpenin A5 was purchased from Santa Cruz Biotechnology, Dallas, Texas.

### Cell culture and reagents

DLD-1 Tet-Off POX cell (DLD-POX) generation has been previously described and were cultured with modification (Liu et al. 2005). The cells were maintained in DMEM containing 5 mM glucose (Life Technologies, Inc., Grand Island, NY) supplemented with 10 % fetal bovine serum (HyClone Laboratories, Logan, UT), 2 mM Glutamax, 0.4 mg/ml G418, and 0.25 mg/ml hygromycin B (Life Technologies), and 20 ng/ml DOX (Sigma).

### Mouse liver mitochondria isolation

Mouse liver mitochondria were prepared in the method of Chappell and Hansford (Birnie 1972). In brief, freshly harvested livers were minced and washed 3× in ice-cold sucrose buffer (0.25 M sucrose, 3.4 mM tris–HCl, 1 mM EDTA, pH 7.4). Liver tissue was homogenized by 5 passes in a Dounce homogenizer. Homogenates were then centrifuged once at 478×*g* for 10 min, and the supernatant centrifuged at 10,000×*g* for 7 min. The pellet was washed with 25 ml ice-cold sucrose buffer and centrifuged 4 times at 10,000×*g* for 7 min, then resuspended in 3 ml ice-cold sucrose buffer. Protein concentration was determined using a BCA kit following the manufacturer’s instructions (Life Technologies).

### Measurement of PRODH/POX catalytic activity

PRODH/POX activity was measured as previously described, with minor modification (Pandhare et al. 2009). After treatment, cells were washed with cold PBS and harvested by trypsinization. Cells were resuspended in cold sucrose buffer [0.25 M sucrose, 3.5 mM Tris, and 1 mM EDTA (pH 7.4)] containing 1× protease inhibitor cocktail 1 and 2 (Sigma) and then sonicated for 20 s at a setting of 20 % (Branson Sonifier 450; Branson Ultrasonics Corp., Danbury, CT). Total protein was determined using the BCA protein assay (Pierce). A 1 ml reaction mixture containing 0.1 M KPO_4_, pH 7.2, 0.12 mg/ml *o*-aminobenzaldehyde (OAB), 0.012 mg/ml cytochrome C, 5 mM proline, and cell extract containing 50–100 μg protein was incubated for 20–60 min at 37 °C. The reaction was terminated by addition of 20 μl of OAB (10 mg/ml in 6 N HCl). The samples were centrifuged and the absorbance of the OAB–P5C complex was measured at 440 nm. A standard calibration curve was generated using purified P5C.

### Oxygen consumption rate assays

XF24 cell culture plates were seeded with 5 × 10^5^ cells and allowed to attach overnight. For PRODH/POX expression, cells were washed 3× with PBS and plated in DMEM containing the indicated amount of DOX and media treatment. One hour prior to the assay, cells were washed 2× with PBS and plated in Seahorse assay media containing 5 mM glucose and 2 mM glutamine, and adjusted to pH 7.4. Cells were incubated for 1 h at 37 °C in a no CO_2_ incubator. Assay reagents (DMSO, oligomycin, FCCP, rotenone, and antimycin A) were loaded at a final concentration of 2.5 µM. Seahorse calibration, mitochondrial fitness tests, and oxygen consumption measurements were performed as per manufacturer’s instructions (Seahorse Biotechnology, Massachusetts).

### Measurement of reactive oxygen species

Cells were washed 3× with Dulbecco’s PBS (DPBS), then placed in DPBS containing 5 µM of 5-(and-6)-chloromethyl-2′,7′-dichlorodihydrofluorescein diacetate, acetyl ester (CM-H_2_DCFDA; Life Technologies). Cells were exposed to the dye for 20 min in a 37 °C incubator. The fluorescence intensity was determined on an adherent cell laser cytometer (ACAS; Meridian Instruments, Inc. Okemos, MI) using 488 nm excitation and 560 nm fluorescence detection. Quantitation was based on total protein measured per well using the BCA protein quantitation kit as per manufacturer’s protocol (Life Technologies).

### Measurement of electron transfer

Catalytic activity of SDH and PRODH/POX was measured using 2,6-dichlorophenolindophenol (DCIP) in the method of Hatefi and Stiggall with modification (Hatefi and Stiggall 1978). 50–100 µg of mouse mitochondria was suspended in 1 ml assay buffer containing 2 mM KCN, 20 mM KPO_4_, 50 µM DCIP, 100 µM CoQ1, and either 10 mM methyl-succinate or proline. Reactions were incubated at 37 °C for 10 min. Changes in absorbance at 600 nm were calculated versus negative control and blank.

### Crosslinking and co-immunoprecipitation

Mitochondrial proteins were cross-linked using dithiobis(succinimidylpropionate) (DSP) according to manufacturer’s instructions (Thermo Fisher Scientific; Waltham, MA). Briefly, 200 mg of mouse mitochondrial fractions was washed 3× in ice-cold PBS. Mitochondria were suspended in cold PBS containing 2.5 mM of DSP and incubated on ice for 2 h. Reaction was quenched by addition of 50 mM Tris–HCl. Immunoprecipitation of Complex II was performed using the monoclonal Complex II immunocapture antibody (Abcam, Cambridge, MA) following manufacturer’s protocol. Mitochondria were solubilized by the addition of 1 % lauryl maltoside (LM) and 1× protease inhibitor cocktail 1 and 2 (Sigma Aldrich) and incubated on ice for 30 min. Mitochondria were centrifuged at 14,000×*g* at 4 °C for 10 min to pellet insoluble material. Lysates were incubated with either 10 µg of Complex II antibody, PRODH monoclonal antibody (A-11; Santa Cruz Biotechnology), or mouse IgG control (Abcam) on ice for 1.5 h. 50 µl of a 50 % slurry of TrueBlot Anti-Mouse IP Beads (Rockland Antibodies, Limerick, PA) was added and samples placed on a rotator overnight at 4 °C. Beads were washed 3× with cold PBS containing 0.1 % LM and suspended in 100 µl of Laemelli SDS-page buffer.

### Western blotting

Cell lysates were prepared and quantified according to established methods. To each well of a 4–12 % or 12 % SDS-polyacrylamide gel, 15–30 μg total protein was applied, electrophoresed, and transferred to nitrocellulose membrane using an iBlot semi-dry transfer apparatus (Life Technologies). Membranes were blocked using Tris-buffered saline with 5 % nonfat milk (pH 7.6; Sigma). Primary antibodies used in this study were SDHA, SDHB, Histone H3 (Abcam), PRODH/POX, NDUFA10, Complex III Rieske FeS (Santa Cruz), Dimethyl Histone H3 (K4), Dimethyl Histone H3 (K36), COX IV (Cell Signaling, Danvers MA), β-actin (Novus Biologicals, Littleton, CO), and subsequently by a secondary anti-mouse/anti-rabbit IgG antibody conjugated to horseradish peroxidase (Jackson ImmunoResearch, West Grove, PA). All blots were washed in Tris-buffered saline with Tween 20 (pH 7.6; Sigma). Detection was done using an ECL kit (GE Healthcare, Pittsburgh, PA). Signals were quantified using Image Studio Light V5 (LI-COR Biosciences, Lincoln, NE).

### Statistical methods

For analysis of mechanisms of inhibition of POX by SDH inhibitors and succinate, data in this study were evaluated using linear and nonlinear regression analysis, Lineweaver–Burk double-reciprocal plot analysis, and analysis of covariance (ANCOVA). Precise estimates of *V*_max_ and *K*_m_ were estimated using modern nonlinear regression (Bates and Watts 1988) methods. Intercepts (1*/v*0) and slopes (*K*_m_/*V*_max_), which were back-calculated from the *V*_max_ and *K*_m_ estimates obtained from *nonlinear* regression analyses, were found to be virtually identical to intercept and slope estimates obtained from *linear* regression analyses using the double-reciprocal Lineweaver–Burk strategy. ANCOVA was used to determine statistical equivalence of intercept and slope estimates in connection with inferences regarding competitive or uncompetitive inhibition in the CoQ1, SUCC, and TTFA analyses (N AWaC^2014^; Alvord 2014; Venables et al. 2002). Statistical analyses were performed with the R Statistical Language and Environment (Team RC 2014).

## Results

### Coenzyme Q1 is electron acceptor for PRODH/POX

It was recently shown that recombinant PRODH/POX isolated from *S. Cerevisae* bound directly to CoQ1 and that this was the mechanism by which PRODH/POX fed electrons from proline into the electron transport chain (Wanduragala et al. 2010). In addition, we have previously demonstrated that PRODH/POX catalysis of proline can be used to support ATP generation under conditions of acute nutrient stress (Pandhare et al. 2009).

**Fig. 1:**
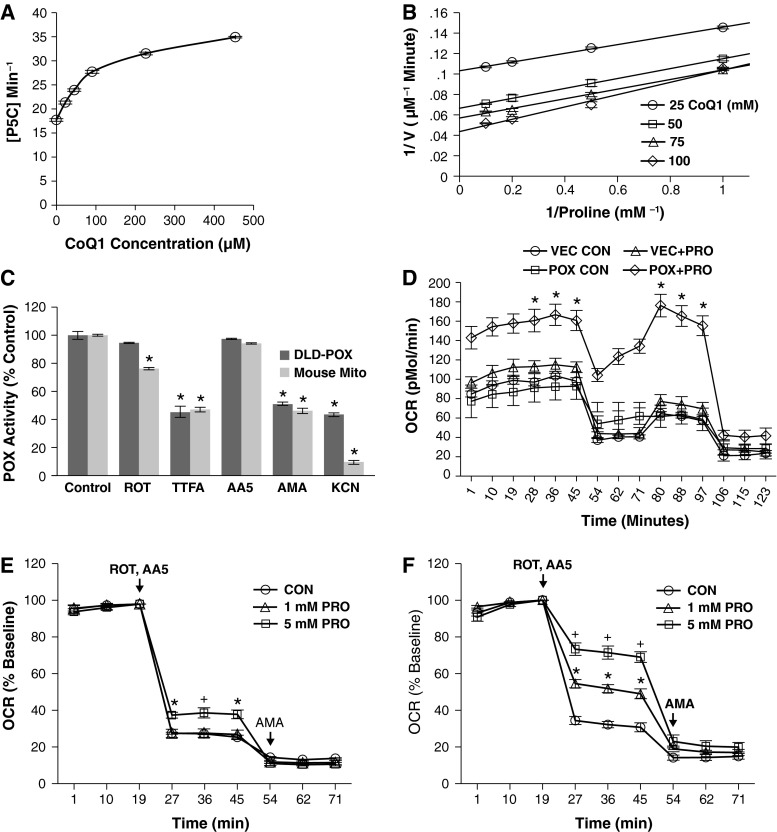
PRODH/POX supports respiration independent of Complex I and II activity. **a** POX activity assay showing the increase in POX activity in the presence of increasing CoQ1. DLD-POX cells were grown in 0.2 ng/ml DOX to allow POX expression. Homogenized cell lysate containing 100 μg protein was incubated in assay buffer with 0, 25, 50, 100, 250, and 500 μM of CoQ1 at 37 °C for 20 min. Absorbance at 440 nm was measured to assess the amount of OAB–P5C complex formed. A P5C standard curve was used to calculate the P5C concentration. Data shown represent mean ± SEM. Compared to CoQ1 = 0, all values are significant to *p* < 0.001. **b** Double-reciprocal analysis of CoQ1-dependent POX activity. DLD-POX lysate with 100 µg protein was incubated with 1, 2, 5, or 10 mM of proline and 25, 50, 75, or 100 µM of CoQ1 in assay buffer at 37 °C for 20 min. Absorbance at 440 nm was used to measure the amount of OAB–P5Ccomplex formed and a P5C standard curve used to calculate the P5C concentration. **c** PRODH/POX activity in DLD-POX lysates and mouse mitochondria show similar dependence on ETC function. PRODH/POX-containing lysates (200 µg protein) or mouse mitochondria (50 µg protein) were incubated in assay buffer at 37 °C for 30 min. Sensitivity of PRODH/POX activity to ETC function was measured by the addition of 50 µM ROT, 2 mM TTFA, 5 µM of AA5, 100 µM of AMA, and 1 mM KCN. Data shown represent mean ± SEM. *p < 0.01 compared to its respective control. **d** PRODH/POX expression is required for proline to support respiration during acute nutrient stress. DLD-VEC control cells (VEC) and DLD-POX cells (POX) were grown in 0.2 ng/ml DOX for 48 h. Cells were incubated in glucose-free media alone (CON) or with the addition of 5 mM proline (+PRO) for 1 h prior to Seahorse XF24 analysis. OCR was measured and a respiratory profile was established by the addition of 2.5 µM DMSO, OLIGO, FCCP, and ROT/AMA at the indicated time points. *p < 0.01 compared to vector control. **e** Seahorse XF24 oxygen consumption analysis of DLD-POX control cells. Cells were grown in 20 ng/ml DOX to suppress PRODH/POX expression. 1 h prior to analysis, cells were incubated in assay media containing 5 mM glucose and either 0 (CON), 1, or 5 mM proline (PRO). OCR was measured at the indicated timepoints. ROT and AA5 were added at 2.5 uM to inhibit Complex I and Complex II activity, respectively. AMA was added at 2.5 μM to inhibit Complex III. Compared to control, *p < 0.01; ^+^ *p* < 0.02. **f** Experimental details as described for (**e**), except in this case, DLD-POX cells were grown in 0.2 ng/ml DOX for 48 h prior to assay. Compared to control, ^+^ *p* < 0.001; *p < 0.01. The following abbreviations apply: *AA5* atpenin A5, *AMA* antimycin A, *CoQ1* coenzyme Q1, *DMSO* dimethyl sulfoxide, *DOX* doxycycline, *FCCP* carbonyl cyanide *p*-trifluoromethoxyphenylhydrazone, *KCN* potassium cyanide, *OAB* 2-aminobenzaldehyde, *OCR* oxygen consumption rate, *OLIGO* oligomycin, *PRODH/POX* proline oxidase, *ROT* rotenone, *TTFA* 2-thenoyltrifluoroacetone

To assess PRODH/POX utilization of CoQ1 as an electron acceptor in our DLD-POX cell model, we added increasing amounts of CoQ1 to DLD-POX lysates and monitored PRODH/POX catalytic activity. As shown in Fig. 1a, PRODH/POX activity increased with increasing CoQ1 in a dose-dependent and saturable manner. These data indicate that PRODH/POX utilizes CoQ1 as an electron acceptor in the oxidation of proline to P5C.

To further characterize its interaction with CoQ1, we performed PRODH/POX activity assays using increasing concentrations of proline and CoQ1 to generate a Lineweaver–Burk plot (Fig. 1b). Under our experimental conditions, the *V*_max_ and *K*_m_ values were 9.72 ± 0.35 µM P5C min^−1^ and 0.42 ± 0.098 mM proline, respectively, using 25 µM of CoQ1. The *V*_max_ and *K*_m_ increased to 21.97 ± 0.47 µM P5C min^−1^ and 1.2 ± 0.09 mM proline, respectively, using 100 µM CoQ1. In a global analysis of covariance of the four data sets, there was no significant difference in the slopes at the *α* = 0.01 level of confidence, *p* = 0.011. This indicates that PRODH/POX binds directly to CoQ1 without the formation of a tertiary complex, consistent with recent reports (Wanduragala et al. 2010).

To determine whether PRODH/POX activity was dependent on ETC function, we compared PRODH/POX activity in DLD-POX cell lysates and mouse mitochondria in the presence of ETC inhibitors. We examined PRODH/POX activity in the presence of CoQ1 alone or in combination with rotenone (ROT), an inhibitor of Complex I, 2-thenoyltrifluoroacetone (TTFA) or atpenin A5 (AA5), inhibitors of Complex II, antimycin A (AMA), an inhibitor of Complex III, or potassium cyanide (KCN), an inhibitor of Complex IV. In both isolated mitochondria and DLD-POX cell lysates, addition of ROT only modestly affected PRODH/POX activity, whereas addition of TTFA significantly reduced PRODH/POX activity (Fig. 1c). This was not the case with AA5, which had no effect on PRODH/POX activity. When AMA or KCN was added to the reaction, PRODH/POX catalytic activity was dramatically reduced. Thus, in both DLD-POX cells and mouse mitochondria, the transfer of electrons from proline to CoQ1 by PRODH/POX was dependent on downstream electron transfer from CoQ1 to Complexes III and IV.

Acute expression of PRODH/POX during nutrient stress led to PRODH/POX- and proline-dependent ATP generation and cell survival (Pandhare et al. 2009). To determine whether PRODH/POX supported oxidative respiration during acute nutrient stress, we incubated either DLD-VEC cells, containing the vector construct, or DLD-POX cells, grown in 0.2 ng/ml doxycycline (DOX) to allow PRODH/POX expression, for 48 h in proline-free media. We then placed the cells in glucose-free media with or without the addition of 5 mM proline for 1 h, and measured cellular oxygen consumption rate (OCR). Whereas the DLD-VEC cells did not respond to proline treatment, the DLD-POX cells were able to support a stable respiratory profile (Fig. 1d).

For additional evidence that PRODH/POX could support respiration solely through proline oxidation, we incubated induced and uninduced DLD-POX cells in proline-free media for 48 h, and then in media containing 0, 1, and 5 mM proline for 1 h, and measured cellular OCR. After measurement of basal respiration, ROT and AA5 were added to eliminate the contributions of Complex I and Complex II to respiration. As shown in Fig. 1e, f, addition of ROT and AA5 reduced respiration to approximately 30 % of normal basal respiration in cells in which PRODH/POX expression was uninduced. The addition of 5 mM proline to uninduced control cells resulted in only a slight recovery of respiration (Fig. 1e). In contrast, addition of proline to PRODH/POX-expressing cells restored respiration at both 1 mM (approximately 50 % basal) and 5 mM (approximately 80 % basal; Fig. 1f). Addition of AMA inhibited respiration in all conditions shown in Fig. 1e, f, indicating that the observed oxygen consumption was dependent on Complex III. This data indicates that proline is a PRODH/POX-dependent respiratory substrate during acute nutrient stress and that it acts independently of Complex I and Complex II but requires functional Complex III.

### Effect of PRODH/POX on respiratory fitness

**Fig. 2:**
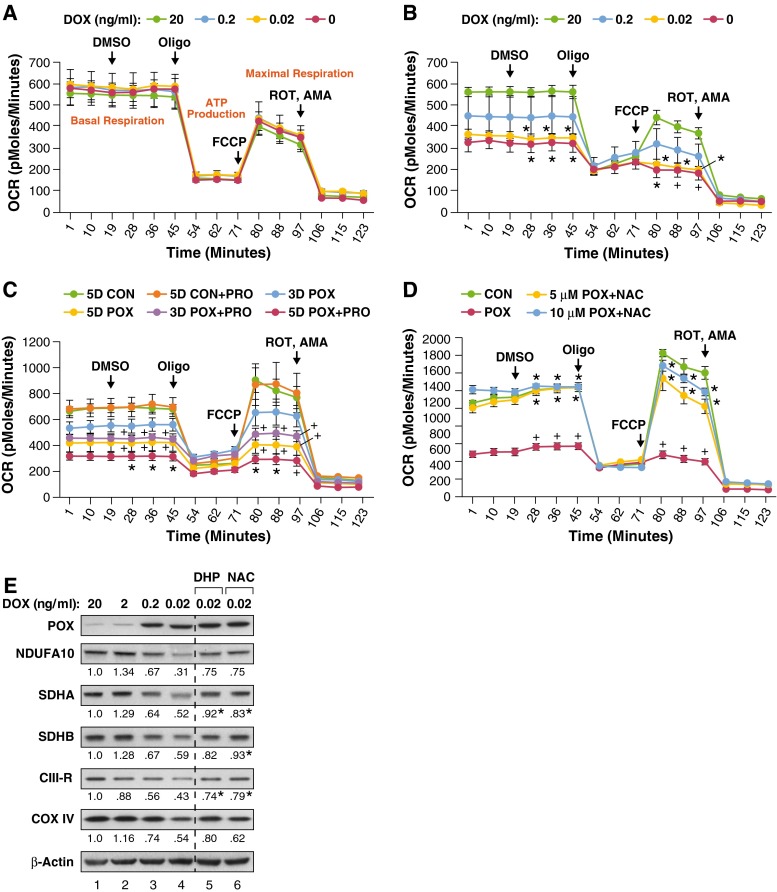
Extended PRODH/POX ROS production decreases total respiratory fitness. Data for each time point represents mean ± SEM (*n* = 3) for all panels. **a** DOX concentration does not affect respiration in DLD-VEC control cells. Cells grown for 48 h in the indicated concentration of DOX. Oxygen consumption rate (OCR) was measured and a cellular respiratory profile was established by the addition of 2.5 µM DMSO, OLIGO, FCCP, and ROT/AMA at the indicated timepoints. **b** Increasing PRODH/POX expression correlates with lower cellular respiration. DLD-POX cells were grown in the indicated amount of DOX and oxygen consumption rate was analyzed as in (**a**). Values with DOX = 0.02 and 0.0 were compared to DOX = 20; *p ≤ 0.01; ^+^ *p* < 0.02. **c** Prolonged PRODH/POX expression and addition of proline correlates with greater decreases in respiration. DLD-POX cells were grown in 0.2 ng/ml DOX to allow PRODH/POX expression for 3 days (3D POX) or 5 days (5D POX) alone or in media supplemented with 5 mM proline (3D POX + PRO and 5D POX + PRO). Respiration by these cells was compared to DLD-POX cells that had been grown for 5 days in 20 ng/ml DOX to suppress PRODH/POX expression, alone or in combination with 5 mM proline (5D CON and 5D CON + PRO, respectively). Oxygen consumption rate was measured and a respiratory profile established as described in (**a**). Values for 3D POX + PRO, 5D POX, and 5D POX + PRO were compared to 5D CON. ^+^ *p* ≤ 0.05; *p < 0.01. **d** Inhibition of PRODH/POX-mediated ROS decreases effects on respiration. DLD-POX cells were grown for 48 h in either 20 ng/ml DOX (CON), or 0.2 ng/ml DOX alone (POX) or in combination with 5 or 10 mM  of *N*-acetyl-l-cysteine (NAC). Oxygen consumption rate was measured and a respiratory profile established as described in (**a**). Compared to control, *p < 0.001; Compared to POX, ^+^ *p* < 0.01. **e** PRODH/POX expression down-regulated ETC component proteins. DLD-POX cells were grown in the indicated ng/ml concentration of doxycycline (DOX) for 48 h, alone or in combination with 10 mM DHP or NAC. Whole cell lysates were harvested, and protein expression of subunits of Complex I (NDUFA10), Complex II (SDHA and SDHB), Complex III (CIII-R, Reiske Fe-S subunit), and Complex IV (COX IV) were analyzed by Western blotting. β-Actin was used as a protein loading control. The band intensities shown below each panel were quantified using Image Studio, normalized to β-actin control, and expressed as the level relative to untreated control (*lane 1* 20 ng/ml DOX). Values for DHP and NAC were compared to the mean ± STD of the ratios for 0.2 ng/mL DOX (*lane 3* **e** and *lane 2*, Fig. 5a) combined with those for 0.02 ng/ml DOX (*lane 4* **e**) Although the DOX concentration for DHP and NAC treatment was 0.02 ng/mL DOX, the values at 0.02 ng/ml DOX were consistently lower than those at 0.2 ng/ml DOX. Thus, the values used represent a higher distribution for the PRODH/POX-mediated effect on ETC proteins. DHP and NAC increased the values of the ETC proteins, i.e., decreased the effect of PRODH/POX. *Value greater than 2 standard deviations of aforementioned distribution, denoting 95 % confidence limits

Our data indicated that PRODH/POX and proline can support respiration during acute nutrient stress. We investigated the effects of PRODH/POX expression on overall respiratory fitness and capacity. We grew DLD-VEC control cells and DLD-POX cells for 3 days with 20, 0.2, 0.02 and 0 ng/ml of DOX to allow for increasing levels of PRODH/POX expression. Manipulation of DOX concentration in DLD-VEC cells had no effect on respiration (Fig. 2a), but DLD-POX cells showed a decrease in both basal and maximal respiratory capacity that was directly proportional to DOX concentration (Fig. 2b).

We examined the effects on respiration resulting from increasing duration of PRODH/POX expression alone or with the addition of proline to the media. DLD-POX cells were grown in either 20 ng/ml DOX as a negative control or 0.2 ng/ml of DOX to allow PRODH/POX expression for 3 or 5 days in the presence or absence of 5 mM added proline. In control cells, proline alone had no effect on basal or maximal respiration, even after 5 days (Fig. 2c). In contrast, expression of PRODH/POX significantly decreased basal and maximal respiration at both 3 and 5 days, and proline addition exacerbated this effect (Fig. 2c). Thus, PRODH/POX-dependent inhibition of respiration can be modulated by the duration of PRODH/POX expression and the availability of proline.

PRODH/POX is a well-established superoxide generator, and the majority of effects of PRODH/POX on signaling are due to generation of ROS (Liu et al. 2006; D’Aniello et al. 2015; Pang and Curran 2014). We investigated whether effects of PRODH/POX on respiration were due to ROS production. DLD-POX cells were grown in 0.2 ng/ml DOX for 3 days with 5 mM proline alone or with the addition of 5 or 10 mM of the antioxidant *N*-acetyl-l-cysteine (NAC). Expression of PRODH/POX with proline showed a dramatic suppression of oxidative respiration, and NAC mitigated their inhibitory effects on and preserved both basal and maximal respiratory capacity (Fig. 2d). These results demonstrate that basal and maximal respiratory capacity are modulated by levels of PRODH/POX induction and proline availability, and that this effect is mediated by ROS generation.

As shown in Fig. 2, PRODH/POX down-regulates cellular respiration through ROS production. We examined the effects of PRODH/POX-dependent ROS on ETC component proteins. To correlate changes in proteins to functional effects on respiration, we grew DLD-POX cells in 20, 2, 0.2, and 0.02 ng/ml of DOX and monitored ETC proteins at increasing levels of PRODH/POX expression (Fig. 2e, lanes 1–4, respectively). In addition, cells were incubated in 0.02 ng/ml of DOX together with either the PRODH/POX inhibitor dehydroproline (DHP) (Fig. 2e, lane 5) or NAC (Fig. 2e, lane 6). To assess statistical significance of DHP and NAC, we calculated mean ± standard deviation of the values for 0.2 and 0.02 ng/ml DOX from Figs. 2e and 5a. Increasing PRODH/POX expression decreased levels of the NDUFA10 subunit of Complex I, SDHA and SDHB, the Reiske subunit of cytochrome C reductase (Complex III), and subunit IV of cytochrome C oxidase (COX IV, Complex IV). Importantly, SDHA, SDHB and CIII-R were restored by either NAC or DHP or by both (Fig. 2e, lanes 5 and 6, respectively. See legend to Fig. 2e). Compared to the POX levels, the values for DHP and NAC indicated by an asterisk were outside of 2 standard deviations from the mean of the POX controls. Taken together, these results provide preliminary evidence that PRODH/POX modulates respiration through ROS-mediated down-regulation of ETC component proteins.

### PRODH/POX is inhibited by Complex II inhibitors and succinate

**Fig. 3:**
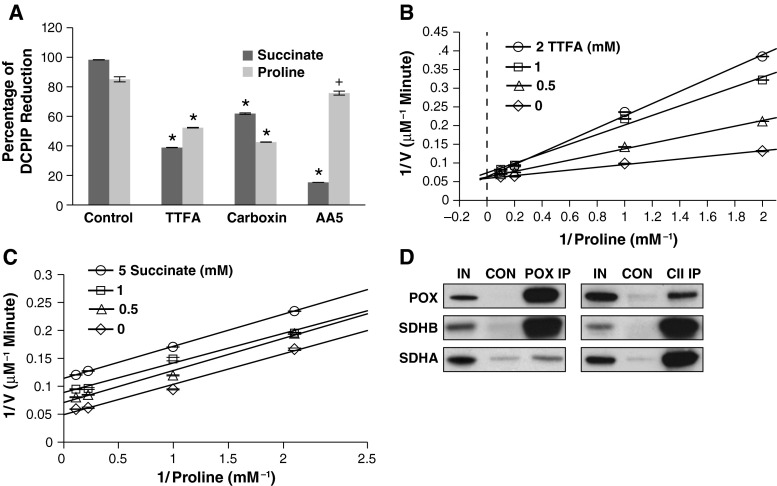
PRODH/POX activity is regulated by succinate and SDH inhibitors and co-localizes with SDH on the mitochondrial inner membrane. **a** PRODH/POX and SDH share similar sensitivity to SDH inhibitors except AA5. Mouse mitochondria (440 µg protein) were incubated in reaction buffer containing either 10 mM of succinate or proline alone (Control) or in the presence of 1 mM TTFA, 1 mM Carboxin, or 5 µM AA5 at 37 °C for 10 min. Absorbance was measured at 600 nm and results calculated as a percent of unreacted control dye. Data shown represent mean ± SEM (*n* = 3) of comparisons against their respective controls. *p < 0.001; ^+^ *p* < 0.05. **b** Double-reciprocal analysis of TTFA-dependent inhibition of PRODH/POX activity. DLD-POX lysate containing 100 µg protein was incubated with 1, 2, 5, or 10 mM of proline and 0, 0.5, 1 and 2 mM of TTFA in assay buffer at 37 °C for 20 min. Absorbance at 440 nm was used to measure the amount of OAB–P5C complex formed and a P5C standard curve used to calculate the P5C concentration. **c** Double-reciprocal analysis of succinate-dependent inhibition of PRODH/POX activity. DLD-POX lysate containing 100 µg protein was incubated with 1, 2, 5, or 10 mM of proline and 0, 0.5, 1, and 5 mM of succinate in assay buffer at 37 °C for 20 min. Absorbance at 440 nm was used to measure the amount of OAB–P5Ccomplex formed and a P5C standard curve used to calculate the P5C concentration. **d** Co-immunoprecipitation of PRODH/POX and Complex II. 4 mg of mouse mitochondria was cross-linked with DSP, then solubilized and incubated with either PRODH/POX (POX IP) or Complex II antibody (CII IP). Control samples were incubated with an equal concentration of a non-specific mouse IgG (CON). Samples were incubated with beads coated with anti-mouse IgG overnight. Cross-linker was cleaved and proteins solubilized with SDS-PAGE buffer. Control and IP lysates were immunoblotted versus a 5 % input control (IN) using PRODH/POX, SDHA, and SDHB antibodies. Data is representative of three separate experiments

We showed in Fig. 1c that TTFA inhibited PRODH/POX activity whereas AA5 did not. The crystal structure of SDH with bound TTFA or AA5 has been solved; both are competitive inhibitors of the ubiquitin-binding site, with AA5 binding at a site more deeply embedded in the enzyme’s catalytic site (Sun et al. 2005; Miyadera et al. 2003; Horsefield et al. 2006). To compare the inhibitory potency of TTFA and AA5 for PRODH/POX versus SDH, we performed enzyme activity assays with either proline or succinate as the substrate. Carboxin, an additional competitive inhibitor of the ubiquitin-binding site of SDH (Ruprecht et al. 2009), was also tested. Both TTFA and carboxin inhibited SDH and PRODH/POX activity with similar efficacy, with TTFA inhibiting 60 % and 35 % and carboxin inhibiting 36 and 42 % of SDH and PRODH/POX activity, respectively. In contrast, AA5 was selective toward SDH, inhibiting its activity by 83 % whereas it only inhibited PRODH/POX activity by 9 % versus control (Fig. 3a).

To elucidate the mechanism by which TTFA inhibited PRODH/POX activity, we performed POX activity assays to construct a Lineweaver–Burk plot (Fig. 3b). *V*_max_ was only modestly decreased, being 17.2 ± 0.4 µM P5C min^−1^ for control and 16.4 ± 0.3 µM P5C min^−1^ with the addition of 2 mM TTFA. In contrast, 2 mM TTFA increased K_m_ from 0.65 ± 0.05 mM proline to 2.7 ± 0.35 mM proline. Statistical tests were performed for the equivalence of the *y*-intercepts for TTFA data pairs. All *p* values were greater than 0.05, indicating that the *y*-intercepts for TTFA data were statistically equivalent. Together, these results suggest that TTFA acts as a competitive inhibitor of PRODH/POX.

We compared the mechanism by which succinate inhibits PRODH/POX activity. We again performed PRODH/POX activity assays to construct a Lineweaver–Burk plot (Fig. 3c). Addition of 5 mM succinate decreased both *V*_max_ and *K*_m_ from 19.1 ± 0.4 to 8.8 ± 0.3 µM P5C min^−1^ and 1.1 ± 0.08 mM proline and 0.55 ± 0.09 mM proline, respectively, versus control. In a global analysis of covariance of the four experimental data sets, there was no significant difference in the slopes at the *α* = 0.05 level of confidence, *p* = 0.75. This indicates succinate inhibits PRODH/POX activity through uncompetitive inhibition.

To examine whether PRODH/POX and SDH could be physically associated on the mitochondrial inner membrane, we performed cross-linking and co-immunoprecipitation assays. We incubated mouse mitochondria with the protein cross-linker DSP, dissolved the mitochondria with 1 % laurel maltoside and immunoprecipitated using an antibody versus either PRODH/POX or Complex II. We then immunoblotted for PRODH/POX and the A and B subunits of SDH. Immunoprecipitation of PRODH/POX resulted in co-immunoprecipitation of both SDHA and SDHB. In addition, PRODH/POX co-immunoprecipitated with Complex II when an anti-Complex II was used (Fig. 3d). These data indicate that PRODH/POX and Complex II co-localize on the surface of the mitochondrial matrix membrane within the length of the DSP spacer arm (12 angstroms).

### Succinate inhibits PRODH/POX-dependent ROS generation

**Fig. 4:**
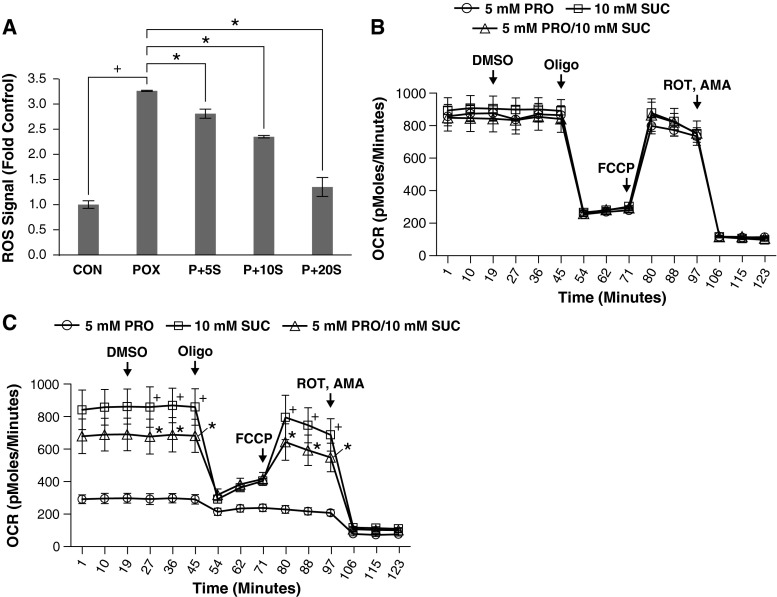
Succinate inhibits PRODH/POX ROS production and effects on respiration. **a** Succinate inhibits ROS production by PRODH/POX-expressing cells. DLD-POX cells were grown for 48 h in 0.2 ng/ml DOX to allow for PRODH/POX expression alone (POX+) or in media supplemented with 5, 10, or 20 mM succinate (P + 5S, P + 10S, and P + 20S). Control wells contained DLD-POX cells grown in 20 ng/ml to suppress PRODH/POX expression (CON). Cells were treated with DCFDA in DPBS for 20 min and fluorescence measured at 488 absorption/530 emission. Signals were normalized by BCA quantitation of protein levels in each well. Comparisons under brackets, *p < 0.01. **b** Succinate does not affect respiration in DLD-VEC control cells. Cells grown for 48 h in 0.2 ng/ml of DOX in media supplemented with 5 mM proline (PRO), 10 mM succinate (SUC), or both (PRO/SUC). OCR was measured and a respiratory profile was established by the addition of 2.5 µM DMSO, OLIGO, FCCP, and ROT/AMA at the indicated timepoints. **c** Succinate inhibits the decrease in respiration induced by PRODH/POX and proline. DLD-POX cells were grown for 48 h in 0.2 ng/ml of DOX to allow PRODH/POX expression. Oxygen consumption rate (OCR) was measured and a respiratory profile was established as described for (**b**). Comparison of 10 mM succinate to 5 mM PRO, ^+^ *p* < 0.01. Comparison of 5 mM POX to 5 MM POX + 10 mM SUC, *p < 0.05. Data represents mean ± standard error of the mean (*n* = 3) for all panels

Our data suggested that PRODH/POX and proline control mitochondrial respiration through ROS-mediated down-regulation of ETC proteins. In addition, our data showed that succinate inhibits catalytic activity of PRODH/POX. We were intrigued whether succinate would modulate the PRODH/POX effect on the ETC. DLD-POX cells were grown in 0.2 ng/ml of DOX to allow PRODH/POX expression, alone or in combination with 5, 10, or 20 mM of methyl-succinate. Cells were then treated with general ROS indicator 5-(and-6)-chloromethyl-2′, 7′-dichlorodihydrofluorescein diacetate, acetyl ester (CM-H_2_DCFDA) to detect total cellular ROS. PRODH/POX expression resulted in a threefold increase in detectable ROS generation versus uninduced control; this ROS generation was inhibited by succinate in a dose-dependent manner (Fig. 4a).

To determine whether succinate inhibition of PRODH/POX protected against the PRODH/POX-dependent down-regulation of respiration, we grew DLD-VEC and DLD-POX cells in media containing proline alone or together with methyl-succinate. DLD-VEC control cells showed no change in basal and maximal respiration whether treated with proline, methyl-succinate, or a combination of both (Fig. 4b). In contrast, PRODH/POX-expressing cells showed a marked reduction of respiration in response to proline treatment that was inhibited by co-treatment with methyl-succinate (Fig. 4c). Taken together, this data indicates that succinate inhibits the effects of PRODH/POX and proline on respiration through the inhibition of PRODH/POX- and proline-derived ROS.

### Succinate inhibition of PRODH/POX restores levels of ETC component proteins

**Fig. 5:**
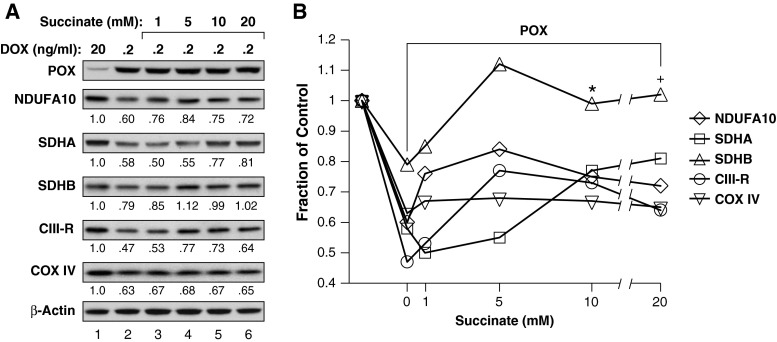
Succinate inhibits the effects of PRODH/POX on ETC component proteins. DLD-POX cells were grown in the indicated ng/ml concentration of DOX for 48 h, alone or in combination with increasing concentrations of succinate as suggested by the effects of succinate on ROS (Fig. 4a). **a** Whole cell lysates were harvested, and protein expression of PRODH/POX and of subunits of Complex I (NDUFA10), Complex II (SDHA, SDHB), Complex III (CIII-R, Reiske Fe-S subunit), and Complex IV (COX IV) was analyzed by Western blotting. The band intensities shown below each panel were quantified using Image Studio, normalized to β-actin control, and expressed as the level relative to untreated control (*lane 1* 20 ng/ml DOX). **b** Since succinate decreased PRODH/POX-mediated ROS in a dose-dependent manner (Fig. 4a) and the levels of proteins in **a** suggested a succinate concentration effect, we constructed a combination plot of protein levels as the fraction of control against succinate concentrations (see text). For the value with treatment by POX (0.2 ng/ml DOX) without succinate, we combined the values from **a** with those obtained in Fig. 2e. We then compared the values statistically. The value with 10 mM succinate was different from that without succinate, *p = 0.020. At 20 mM succinate, the difference was borderline significant, ^+^ *p* = 0.055

Because succinate inhibited PRODH/POX-dependent ROS generation, we investigated whether succinate treatment could modulate PRODH/POX-mediated down-regulation of ETC proteins. DLD-POX cells were grown in either 20 ng/ml DOX or 0.2 ng/ml DOX to allow PRODH/POX expression alone or in combination with increasing concentrations of succinate. To link effects on ETC proteins with effects on ROS (Fig. 4a), we used increasing concentrations of succinate. Expression of PRODH/POX resulted in down-regulation of subunits of Complexes I–IV of the ETC (Fig. 5, lane 1 vs lane 2). Addition of increasing concentrations of succinate resulted in restoring protein levels for Complexes I–III (Fig. 5, lanes 3–6). Levels of the COX IV subunit of Complex IV were not restored by succinate treatment, but it was the only exception of all of the ETC subunits examined.

Since both the decrease in PRODH/POX-generated ROS and the mitigation of the PRODH/POX-mediated decrease in ETC protein levels appeared to be dependent on the concentration of succinate, we plotted the individual ETC proteins versus succinate and showed a general concentration dependence (Fig. 5b). This suggested that succinate-dependent inhibition of PRODH/POX was a common mechanism. With this hypothesis, we compared the values for ETC proteins in combination. Furthermore, we included the values for ETC proteins at 0.2 ng/ml DOX from Fig. 2e. These values represent mean ± STD of ETC proteins. With SUC = 0, 0.635 ± 0.091, *N* = 10, SUC = 10 mM, mean = 0.782 ± 0.122, *N* = 5, different from SUC = 0 with *p* = 0.0203; SUC = 20 mM, values were 0.768 ± 0.156, *N* = 5, different from SUC = 0, with *p* = 0.0546. These values and statistical differences support our working hypothesis of a common mechanism underlying the changes in ETC proteins.

## Discussion

In this study, we have examined the interaction of PRODH/POX with Coenzyme Q1 and with the ETC. While PRODH/POX supports respiration in the short term, PRODH/POX expression results in a decrease of total cellular respiration through down-regulation of proteins of the ETC. Most interestingly, the effects of PRODH/POX on respiration and the ETC can be inhibited by succinate, resulting in a potential regulatory loop.

Our analysis of the relationship between PRODH/POX and CoQ1 suggested that POX can transfer proline-derived electrons directly to CoQ1 without the formation of a tertiary complex. In addition, selective inhibitors indicated that PRODH/POX did not require the activity of Complex I or Complex II to support respiration. This is consistent with studies in other diverse model systems of PRODH/POX activity, including *Z. meas* (corn) mitochondria, purified *S. cerevisiae* PRODH/POX, and PutA from *E. coli* (Elthon and Stewart 1982; Wanduragala et al. 2010; Moxley et al. 2011; Abrahamson et al. 1983). In addition, PRODH/POX dependence on a functional ETC in our DLD-POX expression system was almost identical to that of isolated mouse mitochondria. Thus, we believe our DLD expression system accurately reflects the relationship between PRODH/POX and the ETC.

PRODH/POX was necessary and sufficient to support proline-dependent oxidative respiration during acute nutrient stress, consistent with previous publications showing its pro-survival role during nutrient stress (Pandhare et al. 2009; Liu et al. 2012). In addition, extended expression of PRODH/POX (48–96 h) resulted in the down-regulation of basal oxidative respiration and maximal respiratory capacity. This effect was dependent on the duration and intensity of POX expression as well as the addition of exogenous proline, and was inhibited by the co-treatment with NAC. Reduction of respiratory capacity correlated with reduction in levels of protein subunits of Complexes I–IV of the ETC, which was reversed by co-treatment of cells with either DHP or NAC. These effects are most readily explained as resulting from proline oxidation and ROS generation by PRODH/POX.

Inhibition of PRODH/POX by TTFA was through competitive inhibition. Both TTFA and carboxin have previously been shown to compete for the ubiquinone-binding site of SDH that is formed when the 4 SDH subunits combine to form the holoenzyme (Sun et al. 2005; Ruprecht et al. 2009). Based on this, one possibility we considered was that PRODH/POX required the ubiquitin-binding site of SDH for its activity. However, AA5, which has been shown to bind deeper in the ubiquinone-binding pocket of SDH (Miyadera et al. 2003; Horsefield et al. 2006), showed very little inhibition of PRODH/POX. In addition, the interaction of PRODH/POX with CoQ1 indicated direct binding to PRODH/POX. We think it more likely that the SDH inhibitors compete for the ubiquitin-binding site on PRODH/POX. The observation that TTFA and carboxin can inhibit PRODH/POX and SDH to a similar degree at the concentrations used may warrant caution in their use as SDH-selective inhibitors.

Although succinate inhibition of PRODH/POX has been investigated (Kowaloff et al. 1977; Norden and Matanganyidze 1974), to our knowledge this is the first study to examine the effects of this inhibition on PRODH/POX-mediated ROS generation and mitochondrial metabolism. Our analysis showed that succinate inhibits POX through uncompetitive inhibition. This mechanism makes it very unlikely that the observed effects are due to non-selective inhibition of PRODH/POX by succinate. Treatment with succinate inhibited PRODH/POX-dependent ROS production and prevented PRODH/POX-dependent ROS effects on oxidative respiration. Succinate also protected ETC component proteins from PRODH/POX ROS-mediated down-regulation with almost the same efficacy as DHP and NAC.

As an uncompetitive inhibitor, the affinity of succinate is for the enzyme-substrate complex of PRODH/POX and proline rather than for the enzyme binding site for proline. Additionally, our data show that PRODH/POX and SDH co-localize on the mitochondrial inner membrane, where local substrate concentrations would have the potential to affect the activities of both enzymes. Thus, in the presence of low levels of proline, higher levels of succinate could act to inhibit PRODH/POX activity and ROS generation. This may provide an additional level of regulation of PRODH/POX stress signaling until cellular levels of TCA cycle intermediates fall below a critical point. The specific role that SDH plays in the transmission of the PRODH/POX-generated ROS signal remains to be elucidated, but a coordinated role with PRODH/POX would be consistent with the observation that SDH-mediated ROS generation was essential for a hypoxia-dependent stress response in mouse lung sections (Paddenberg et al. 2003, 2012) and that Complex II has been found to be a site of ROS generation resulting from proline oxidation (Goncalves et al. 2014).

The data presented here introduce a novel relationship between PRODH/POX, proline, and succinate and the regulation of respiration. Depending on cellular context, this relationship could provide an additional point of regulation between the identified inducers of PRODH/POX and regulation of cellular energy levels and routing of metabolites. More intriguing is the possibility that the proline found in extracellular collagen may serve both as a source of energy for tumors and tissues through proline catabolism, and also as a signaling and metabolic link between the extracellular environment during development and tumor formation. It is becoming increasingly apparent that dysregulation of metabolism and the resulting change in metabolite levels affect not only cell transformation (Ward et al. 2010; Lu et al. 2013), but influence tumor cell aggressiveness and metastatic potential, as has been shown recently in ovarian cancer and ovarian cancer stem cells (Vermeersch et al. 2014, 2015). The role of PRODH/POX as a direct mediator of ROS-dependent signaling and central mediator of metabolic intermediates will, no doubt, continue to play an interesting role in cellular development and transformation.

## Summary

This work shows for the first time the regulation of respiratory fitness by PRODH/POX through downregulation of ETC proteins, and the inhibition of these effects by succinate. The existence of a regulatory loop between PRODH/POX, the ETC, and succinate would link the various stress pathways that induce PRODH/POX with ETC regulation and TCA cycle metabolite flux. Changes in proline and succinate homeostasis may be a mechanism of epigenetic gene regulation during differentiation and tumorigenesis, and this possibility should be investigated further.

## Abbreviations

AA5: Atpenin A5
AMA: Antimycin A
CIII-R: Complex III Rieske subunit
CM-H_2_DCFDA: 5-(and-6)-Chloromethyl-2′,7′-dichlorodihydrofluorescein diacetate, acetyl ester
CoQ1: Coenzyme Q1
COX IV: Cytochrome C oxidase subunit IV
DCIP: 2,6-Dichlorophenolindophenol
DHP: 3,4-Dehydro-l-proline
DMSO: Dimethyl sulfoxide
DOX: Doxycycline
DSP: Dithiobis(succinimidylpropionate)
ETC: Electron transport chain
FCCP: Carbonyl cyanide *p*-trifluoromethoxyphenylhydrazone
NAC: *N*-acetyl-l-cysteine
NDUFA10: NADH dehydrogenase [ubiquinone] 1 alpha subcomplex subunit 10
OAB: 2-Aminobenzaldehyde
OCR: Oxygen consumption rate
OLIGO: Oligomycin
P5C: Pyrroline-5-carboxylate
PRODH/POX: Proline dehydrogenase/proline oxidase
ROS: Reactive oxygen species
ROT: Rotenone
SDHA: Succinate dehydrogenase subunit A
SDHB: Succinate dehydrogenase subunit B
SDH: Succinate dehydrogenase
SUC: Succinate
TTFA: 2-Thenoyltrifluoroacetone
